# Nature-Inspired Designs in Wind Energy: A Review

**DOI:** 10.3390/biomimetics9020090

**Published:** 2024-02-01

**Authors:** Farzaneh Omidvarnia, Ali Sarhadi

**Affiliations:** Department of Wind and Energy Systems, Technical University of Denmark (DTU), Frederiksborgvej 399, 4000 Roskilde, Denmark; asar@dtu.dk

**Keywords:** biomimetics, bio-inspired design, biomimicry, wind energy systems, wind turbine, turbine blade

## Abstract

The field of wind energy stands at the forefront of sustainable and renewable energy solutions, playing a pivotal role in mitigating environmental concerns and addressing global energy demands. For many years, the convergence of nature-inspired solutions and wind energy has emerged as a promising avenue for advancing the efficiency and sustainability of wind energy systems. While several research endeavors have explored biomimetic principles in the context of wind turbine design and optimization, a comprehensive review encompassing this interdisciplinary field is notably absent. This review paper seeks to rectify this gap by cataloging and analyzing the multifaceted body of research that has harnessed biomimetic approaches within the realm of wind energy technology. By conducting an extensive survey of the existing literature, we consolidate and scrutinize the insights garnered from diverse biomimetic strategies into design and optimization in the wind energy domain.

## 1. Introduction

With 3.8 billion years of evolution, nature has developed technologies that rival or outperform those developed by humans [1]. Biomimetics, also known as biomimicry, bio-inspired, or biologically inspired [2], is a burgeoning research field that draws inspiration from natural models, systems, and elements to provide innovative design solutions for various problems [3,4]. The approach aims to integrate designs inspired by biological organisms into engineered technologies. As an interdisciplinary field, biomimetics connects the collaborative efforts of biologists, physicists, chemists, engineers, and architects, offering the potential to deliver sustainable solutions [5], and enables the development of machines that imitate the performance of organisms, especially when their performance surpasses current mechanical technology or offers innovative solutions to existing challenges [6]. The term “biomimetics” was coined by Otto Schmitt in the 1950s to describe the process of drawing inspiration from nature to address practical challenges we face [7]. In 1997, Janine M. Benyus introduced the term “biomimicry” in her book *Biomimicry: Innovation Inspired by Nature* [8].

Biomimetics, drawing inspiration from biological systems, has long intrigued engineers and designers, tracing back to Leonardo da Vinci’s studies of bird flight and even earlier to myths like Daedalus and Icarus. Historical references, including King Solomon’s throne adorned with mechanical creatures and the Talmud’s tales of the Golem, illustrate this enduring fascination [9,10]. Bar-Cohen has compiled a comprehensive review that explores the intricate technological applications of various biological systems in the field of engineering [11]. The fundamental inclusive principles inherent in nature can be broadly categorized into ten key aspects, forming the foundation of bioinspiration [12,13].

Nature’s 10 principles [13] emphasize sustainable and efficient practices. Energy efficiency is key, with only essential energy used for functions. Recycling and reuse transform waste into resources, while resilience and diversity ensure balance amidst chaos. Nature optimizes resource use for equilibrium and fosters collaboration for collective success. Continuous learning and adaptation are achieved through feedback mechanisms. Safe materials and chemicals are used, with a reliance on abundant resources and cautious use of scarce ones. Nature’s adaptability to its environment ensures survival, integrating function and form for minimal energy and material use.

One promising application of biomimetics lies in the design of advanced materials, thanks to their remarkable mechanical, hydrodynamic, optical, and electrical properties, which have evolved over time [10,14,15,16,17,18,19,20]. Interest in biomaterials and biostructures has grown, driving the development of intelligent biological systems, inspired by a variety of notable examples [21,22]. However, the challenge extends beyond imitating nature’s materials and structures; it involves understanding the principles and mechanisms behind biological systems and their functions [23]. Additionally, engineering often faces unique conditions and constraints, potentially resulting in different materials [24,25,26]. Biomimicry-inspired self-healing materials encompass methods from vascular-like systems to nanoparticle-based delivery [27,28]. Techniques in self-healing concrete, like adhesive conduits and shape-memory alloys [29,30], replicate biological materials, resulting in synthetic versions with improved strength and durability, suitable for aerospace, construction, and manufacturing applications.

Biomimetics has significantly influenced the field of robotics and automation [31,32]. By mimicking the locomotion and sensory mechanisms observed in animals and insects, engineers have developed highly agile and efficient robots [33,34,35,36,37].

Biomimetics is revolutionizing sustainable energy technologies, particularly in solar panel and wind turbine design, in response to the growing global energy demand and the surge in renewable energy exploration [38,39]. In the past decade, the field of automobile design has expanded its influence beyond aesthetics, extending to functionality, exemplified by DaimlerChrysler’s bionic concept car inspired by the boxfish’s exterior shape [40]. The Japanese bullet train’s design, inspired by the kingfisher’s beak, reduces sonic booms and air resistance, mirroring the bird’s splash-minimizing dive. This biomimicry enhances the train’s acceleration and energy efficiency, earning it the “bullet train” nickname [40,41,42].

Wind energy has witnessed rapid expansion in recent years. Projections indicate that wind energy could constitute over 40% of all renewable energy sources by 2030 [43] and contribute to fulfilling approximately 20% of the world’s energy requirements [44].

The development of wind turbines and their aerodynamic modeling has been a focal point in recent research, with considerable advancements and reviews documented over the past few decades [45,46,47]. In the realm of biomimetics, Roy et al. [48] contributed significantly to wind turbine research by comprehensively reviewing the application of bio-inspired tubercles on horizontal axis wind turbine (HAWT) blades, highlighting their potential to enhance aerodynamic performance, especially in post-stall regimes and varying wind conditions. Siram et al. [49] reviewed small wind turbines (SWTs), particularly for off-grid and decentralized energy systems, emphasizing their operation under low Reynolds number and tip speed ratio (TSR) conditions. It addresses key aspects like airfoil selection, blade design, and aerodynamic enhancements, including bio-inspired profiles, suitable for low-Re and low-k SWTs.

The present study offers an extensive review of the utilization of biomimetics in the development of wind turbine systems. It studies various natural inspirations applicable across various facets, including blade structure design, component development, and innovative proposals for new wind turbine configurations. The objective of this review is to underscore the potential advantages, inherent challenges, and progressive advancements associated with incorporating nature-inspired solutions in wind turbine design.

## 2. Methodological Approach: A Review of Inspirational Sources and Research Trends

By tracing the trajectory of past research endeavors, the aim was to discern notable patterns based on the sources of biological inspiration that have informed these investigations. In doing so, the endeavor was to shed light on the extent of researchers’ adaptability and creativity in drawing inspiration from the natural world. Additionally, this exploration serves as a foundation upon which we can envision the uncharted frontiers and novel possibilities that lie ahead in the intersection of biomimetics and wind energy research.

**Figure 1:**
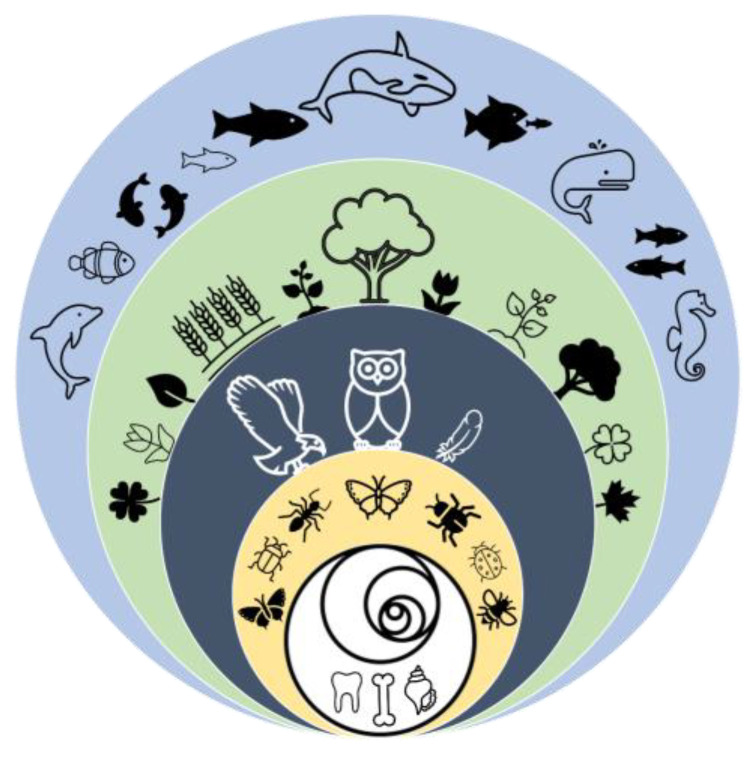
Diverse sources of nature inspirations in wind turbine system design.

Sources were included in this study based on the claims or explanations pertaining to the advantages of bio-inspired design within the literature. A substantial portion of these sources were derived from specialized journals, conferences, and compilations centered on bio-inspired design in the context of wind turbine system design and optimization. The sources authored by the same individual were treated as distinct entities in our analysis. Therefore, the presence of a particular claim across numerous sources may indicate its widespread acceptance or its significance within a select group of prolific authors. The aim was to encompass a wide array of academic publications and authors, ensuring a broad search scope. Consequently, recurring claims found across a diverse set of authors are likely to reflect prevailing trends in the field, although the numerical ratio of sources making a specific claim to the total sources analyzed may not be statistically representative of the entire field. Figure 1 illustrates the investigated categorization of bioinspiration in wind turbine system designs.

## 3. Sources of Inspirations

In this study, focusing on wind energy, the predominant natural sources that have inspired advancements in the field were identified. For ease of understanding, each source is represented by a unique visual symbol (Table 1). The table represents the major and subcategories of major sources of inspiration in this realm.

## 4. Learning from the Plant Kingdom in Wind Energy

### 4.1. Movement of Tree Branches and Leaves

Plants have long served as a profound source of inspiration in the realm of biomimetic engineering, particularly within the context of sustainable energies. One of the most prominent examples is the development of solar photovoltaic technology, which draws inspiration from the way leaves capture sunlight and convert it into chemical energy [50,51]. Engineers have endeavored to replicate the efficiency of photosynthetic systems by designing advanced solar cells that emulate the molecular structures found in plants.

In the pursuit of sustainable energy, biomimicry extends beyond energy generation to energy storage. Researchers are exploring biomimetic solutions for efficient energy storage systems by looking at how plants store and release energy through processes like transpiration and ion transport [52,53]. These innovations hold the promise of more efficient and eco-friendly energy storage solutions.

**Figure 2:**
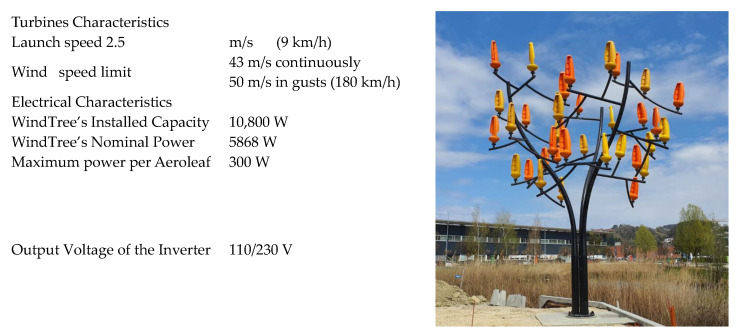
Technical specifications of the Windtree and WindTree is a steel structure (trunk and branches) on which 36 Aeroleaf^®^ were installed. Reprinted with permission from [57], Copyright© 2024, New World Wind.

Plants have also inspired breakthroughs in wind energy [54]. Biomimetics has led to the creation of wind turbine designs inspired by the swaying of palm fronds or the aerodynamic efficiency of leaves [55,56]. The Aeroleaf^®^ [57], a French company, represents a micro wind turbine with a patented design influenced by leaves and trees (Figure 2). Within this design, a synchronous generator with permanent magnets makes up each Aeroleaf. The maximum power per leaf is reported around 300 W.

Within the realm of sustainable energy solutions, novel approaches have gained prominence, including the utilization of tree movement to harvest energy. Harvesting energy from the movement of trees has the potential to provide power for wireless devices deployed in densely wooded environments where other energy sources, such as solar, may be limited. The study by McGarry et al. [58] focused on investigating the amount of energy and power available from the motion of a tree in a sheltered position, specifically through Beaufort 4 winds.

Several methods for extracting energy from tree movement have been explored, including harvesting energy from the tree’s horizontal acceleration, lean angle, and force/displacement, and the force/displacement approach showed the greatest potential for harvesting energy. The tree’s average power output, over 900 s, to lift and lower the mass in only one (arbitrarily selected) direction, was calculated to be 44.7 mW in one axis. According to the results of this study from analysis of tree movement energy harvesting methods, the total wind power dissipated by the tree was around 496 W [58].

**Figure 3:**
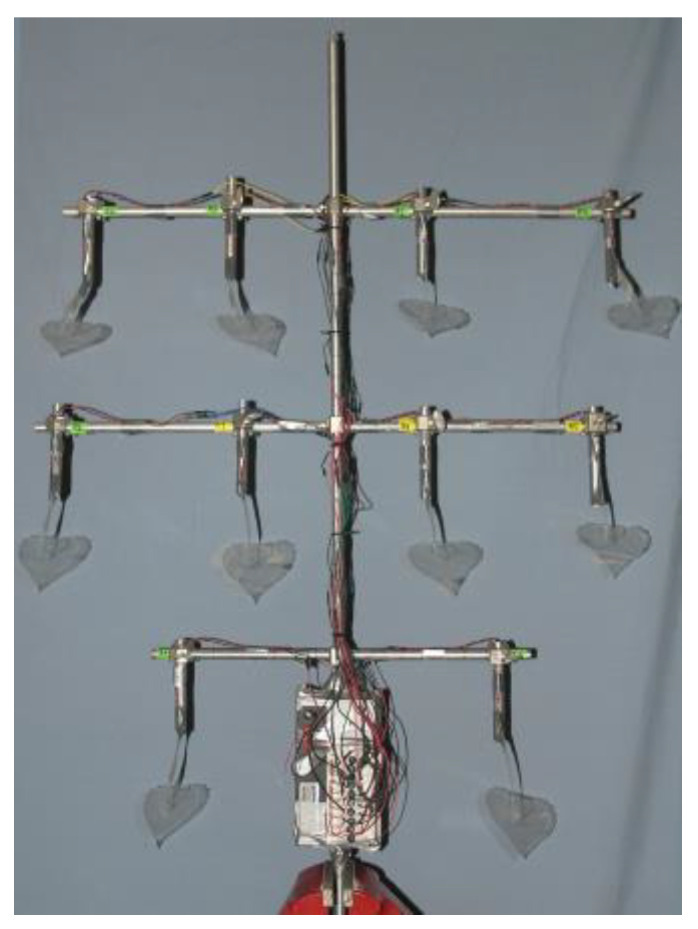
Cottonwood-shaped plastic leaves mounted on an aluminum trellis [59].

McCloskey et al., in their study [59], focused on the potential of using plant-inspired designs to convert wind energy. The goal of their study was to explore alternative methods to traditional wind turbines to overcome their limitations, such as noise, visual impact, and restricted deployment in residential areas. They investigated the use of artificial plants containing piezoelectric elements, which can generate electrical energy from wind-induced vibrations (Figure 3).

### 4.2. Lotus Flower Inspiration

In engineering, the study of flower petals has inspired the development of lightweight and resilient materials. Mimicking the microstructures and properties of petals, researchers have created materials that are not only strong but also flexible, making them ideal for applications in aerospace, automotive, and construction industries [60]. Flowers also influence biomimetic designs in robotics and medical devices. The study of flower-like structures has informed the development of soft robots and medical implants that mimic the flexibility and adaptability of petals, offering solutions for minimally invasive procedures and patient comfort [61,62]. Furthermore, the self-cleaning properties of some flower surfaces, like the lotus leaf, have inspired the creation of superhydrophobic materials. These materials have a wide range of applications, from self-cleaning surfaces to water-resistant coatings on textiles and electronics.

The Nile’s Lotus flower, abundant in Egypt, holds cultural and historical significance [63,64].

**Figure 4:**
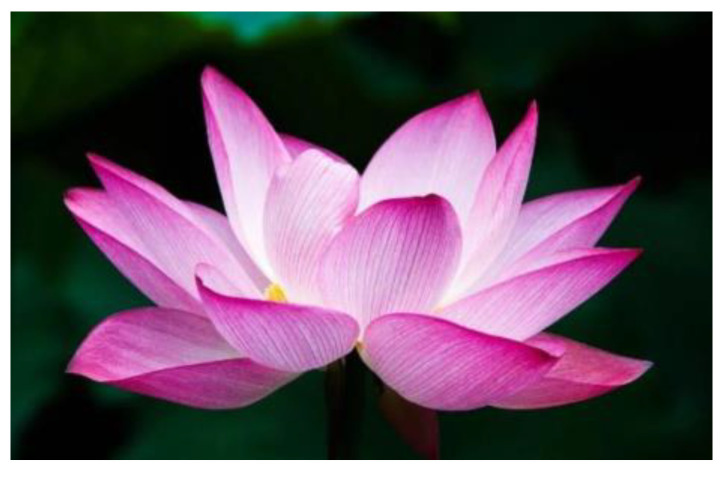
Nelumbo nucifera, also known as sacred lotus, Indian lotus, or simply lotus [63].

Abdelrahman et al. [63] investigated the development of a new design for HAWT blades, drawing inspiration from the flower of Nelumbo nucifera (Sacred lotus, Figure 4). Recognizing the aerodynamically favorable structure of the lotus flower, the study aimed to enhance the efficiency of wind turbine blades by integrating these natural design elements. The authors claimed that the lotus-inspired turbine model exhibited a 31.7% increase in output power compared to the traditional NACA 2412 airfoil turbine model and that the design is suitable for small- and medium-scale wind turbine projects.

### 4.3. Insights from Seeds

**Figure 5:**
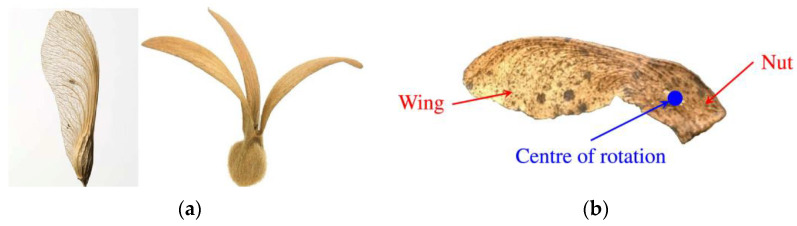
(**a**) Maple seed (**left**) and Triplaris samara seed (**right**). (**b**) Two main parts of a maple samara [74,75]. Reprinted with permission from [75], Copyright ©, 2024, Elsevier.

The maple tree (Acer) is adapted to habitats with poor nutrients in temperate climates [65]. Maples disperse their seeds by wind, updrafts, and turbulent gusts, like many other pioneer trees [66,67]. In windy conditions, maple seeds disperse quickly and start to autorotate within 1 m of detaching from the tree. The wing-shaped seed (Figure 5) autorotates because the heavy nut, and hence the center of gravity, are located at the base [68,69,70,71]. Inertial and aerodynamic properties of maple and other rotary seeds interact to create stable autorotation, which is still poorly understood [68,72,73]. Supposedly, autorotation creates lift to allow seeds to descend for longer periods. Autorotating seeds, despite their small size and slow velocity, generate surprising lift forces according to detailed performance studies [68,69].

The aerodynamic characteristics of auto-rotating maple and Triplaris samara seeds were extensively investigated by Lentink et al. [76]. Their study revealed that these seeds exhibit a notable capacity for generating unexpectedly high lift during their descent. The findings indicate that the “helicopter” seeds of maple trees and other similar autorotating seeds rely on the aerodynamic mechanism of generating lift as they descend slowly through the air. However, the specific means by which this lift generation occurs have remained unclear.

Holden et al. analyzed the flow field around a maple seed as it rotates and draws comparisons to wind turbine blades [77]. Experimental measurements and high-speed video imaging were used to determine the physical values of a real maple seed sample. The power coefficient (C_P_) of the maple seed was found to be 0.59, comparable to the range from 0.45 to 0.48 for many wind turbines and close to the Betz limit of 0.593.

Inspired by the study by Lentink et al. [76], Seidel et al. focused in their study on designing blades that mimic the shape of maple and triplaris samara seeds [78], which are known to generate extra lift due to their geometrical properties.

**Figure 6:**
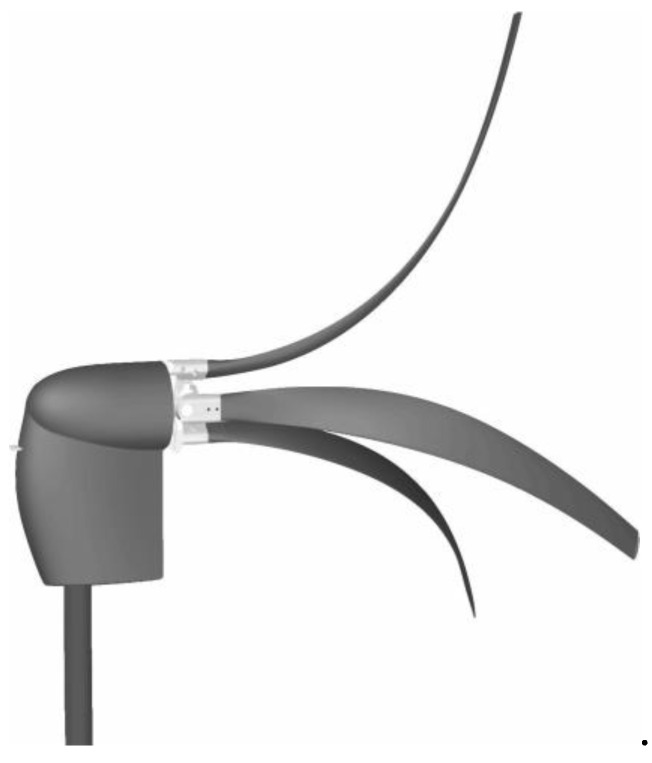
3D model of a bio-inspired wind turbine [78]. Reprinted with permission from [78] Copyright © 2024, Elsevier.

Herrera et al. investigated the structural design and manufacturing process of a low-scale bio-inspired wind turbine blade based on the Triplaris americana tree seed [79]. The blade design was derived from an analysis of the seed’s curvature and airfoil along its wingspan, resulting in a nonconventional HAWT with three blades (Figure 6).

The blade’s geometry and composite structure showed potential for clean energy generation, surpassing commercial wind turbines in terms of C_P_ and energy conversion factor with a peak C_P_ of 0.55 during testing. The authors mention that this bio-inspired design is particularly promising for areas with low wind speeds, offering a cost-effective alternative for electricity generation in developed countries.

Carré et al. [75] present the design and experimental testing of a SWT inspired by the shape and behavior of maple samaras. The blade angles and the number of propeller blades were optimized, and a miniature generator with low-friction ceramic bearings was used for power conversion.

The performance of the 44 mm diameter HAWT was tested under wind speeds ranging from 1.2 to 8 m/s. The electrical power output, measured in resistive load, ranged from 41 μW to 81.7 mW, resulting in an overall efficiency between 2.6% and 17.8%. The C_P_ reached 28.4%, which is among the highest rates in terms of efficiency and power density compared to other miniature wind turbines in the literature.

The samara-based wind energy harvester demonstrates an extended range of airspeeds for energy harvesting, with an operating speed as low as 1.2 m/s. It offers potential applications in areas where regular maintenance is challenging or batteries cannot be used, such as difficult-to-access locations, buildings, and houses.

The biomimicry principles by drawing inspiration from maple samara seeds’ morphology and flight capabilities were explored by Çalışkan et al. [79]. Employing mathematical modeling, the team translated these natural features into a virtual environment, yielding a biomimetic wing model. The wing exhibited a stall angle range of 40°–45°, a substantial improvement compared to conventional wings. Also, the biomimetic wing design demonstrated stability across changing wind velocities, with minimal variations in aerodynamic performance parameters.

**Figure 7:**
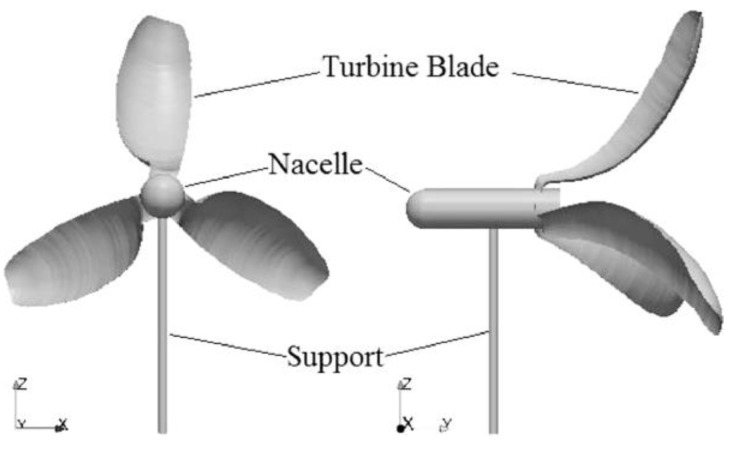
3D models of the proposed biomimetic downwind HAWT inspired by Dryobalanops aromatica seed [80]. Reprinted with permission from [80] Copyright © 2024, Elsevier.

Chu et al. [80] investigated the performance of a rigid biomimetic HAWT rotor blade inspired by the Dryobalanops aromatica seed (Figure 7). They compared the C_P_, thrust coefficient (C_T_), and blade root bending stresses of the proposed biomimetic wind turbine with a tapered and twisted blade from Krogstad and Lund [81]. The simulation results revealed that the biomimetic wind turbine showed a maximum C_P_ of 0.386 at a TSR of 1.5 and a free stream velocity (U ∞) of 10 m/s. It exhibited a better self-starting ability and higher torque compared to the reference turbine.

**Figure 8:**
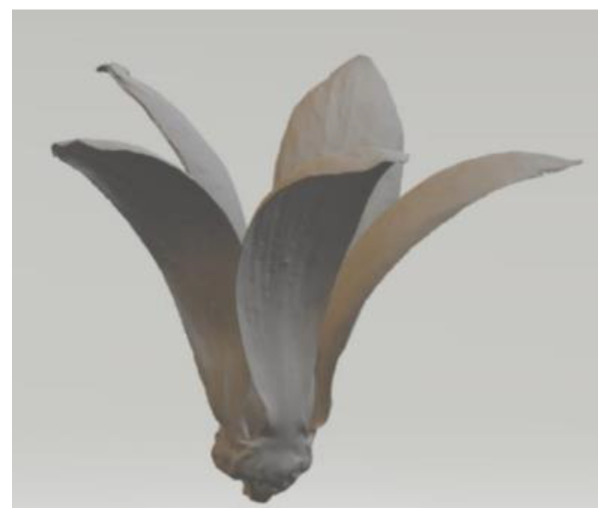
3D-scanned model of the Borneo camphor seed [82].

Chu et al. [82] proposed a new thin-cambered bent biomimetic wind turbine blade design inspired by the 3D geometry of the wing of a Borneo camphor seed (Figure 8). The wings of the Borneo camphor seed exhibit thinness, camber, and bending, which enable autorotation during propagation and slow down the seed’s falling speed. By mimicking these wing characteristics, they proposed a high-performance biomimetic wind turbine design that shares a similar rotating mechanism with the Borneo camphor seed.

In another study Chu et al. [83] conducted a comparative study by examining the performance of a bio-inspired flexible-bladed wind turbine (FBWT) mimicking the wings of a Borneo camphor seed against a traditional rigid-bladed wind turbine (RBWT) at a centimeter scale. The primary objective was to evaluate and contrast various aspects of these wind turbines, including electrical power output, start-up behavior, blade coning, and yawing characteristics.

Regarding electrical power output, the FBWT consistently outperformed the RBWT, yielding a maximum power output of 7.33 mW compared to the RBWT’s 6.52 mW. The FBWT achieved a higher maximum C_p_ of 0.0870, achieved at a TSR of 3.20 and a wind speed of 1.83 m/s, surpassing the RBWT’s 0.0576 at a TSR of 3.56 and a wind speed of 2.04 m/s.

**Figure 9:**
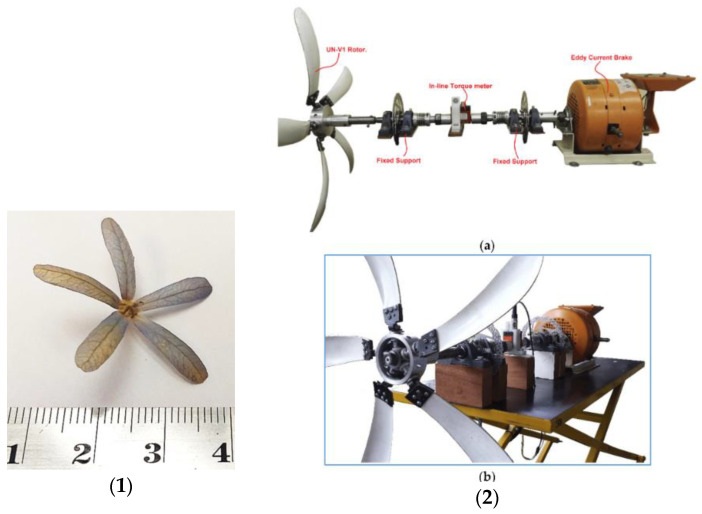
(**1**) Petrea volubilis seed, so-called Queen’s Wreath; (**2**) wind rotor test bench. (**a**) Main parts. (**b**) Isometric view [84].

Gaitan-Aroca et al. [84] investigated a biomimetic wind rotor design inspired by the Petrea volubilis seed in terms of its performance as a HAWT (Figure 9). The biomimetic wind rotor design exhibited vorticity generation and a predominant tangential vortex motion. The C_p_ of the biomimetic wind turbine model reached its highest value which was higher than that of the benchmark case, while the C_T_ at the peak C_P_ for the biomimetic wind turbine cases was lower than that of the benchmark case. 

**Figure 10:**
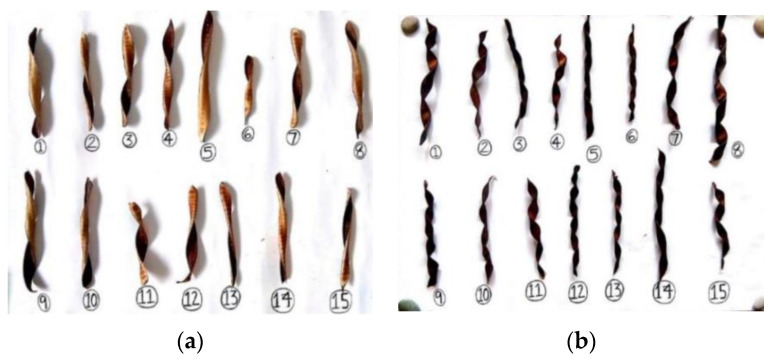
Seed pod specimens of (**a**) Bauhinia variegata and (**b**) Mimosa [85].

Venkataraman et al. conducted a numerical investigation on the stand-still characteristics of a bio-inspired vertical axis wind turbine (VAWT) rotor designed for an urban environment [85]. The rotor shapes were inspired by the seed pods of two commonly found trees in India, Mimosa and Bauhinia variegata (Figure 10). The simulations revealed that a single-bladed helical rotor generated torque of approximately 0.26 Nm at a wind speed of 2 m/s, which exceeded the typical cogging torque of a 500 W permanent magnet generator. 

**Figure 11:**
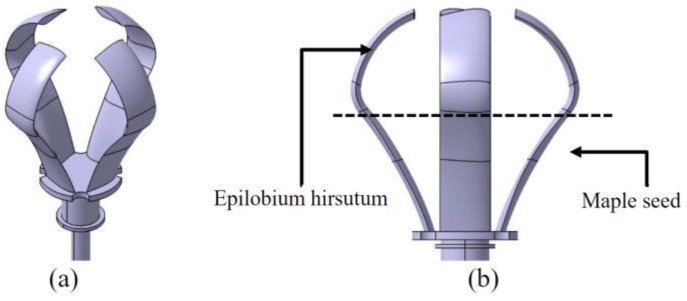
Proposed wind turbine configuration by Ashwindran et al.: (**a**) wind turbine design, (**b**) adapted morphology [86].

Ashwindran et al. [86] studied an unsteady numerical analysis on a novel biologically inspired VAWT designed for offshore regions of Malaysia. The turbine’s blade shape was derived from a hybrid design inspired by the maple seed and Epilobium hirsutum (Figure 11). The turbine exhibited favorable performance at λ = 1.3 and λ = 1.7, yielding C_P_ = 0.245 and C_P_ = 0.262, respectively.

Table 2 presents a summary of studies inspired by plants and their key features, showcasing the research challenges, the advantages of bio-inspired solutions.

## 5. Insect-Inspired Approaches to Wind Energy

Conventional wind turbines are constrained to operate within a narrow operational envelope centered around their optimal working point. This inherent limitation poses challenges when confronted with variable wind conditions, leading to pronounced energy losses and economic inefficiencies.

With the action of an incoming wind, thin elastic structures, like wings and leaves, adjust the torque exerted by the fluid pressure when they bend, changing the balance between external mechanical loads [87,88,89]. During windy conditions, plants bend to reduce drag and avoid damage [90,91,92]. Insects, on the other hand, use this ability to change the direction of pressure forces to increase thrust without adding energy. In the same vein, wind turbine blades can also be made flexible.

### Wing Structure of Cicada, Bee, Wasp, Mosquito, and Dragonfly

Insect wings provide a blueprint for structures that can improve energy harvesting efficacy in wind turbines. The potential of inspiration from insects to design a turbine for enhancing energy generation was explored by Segev et al. [93]. The study presented two designs inspired by different insects, namely the cicada, bee, wasp, mosquito, and dragonfly, and compared their energy outputs to a traditional control design. Insect wings serve as a source of inspiration due to their ability to minimize drag and increase efficiency through instantaneous adjustments during flight. The biomaterial, wing-inspired designs in this study successfully increased the RPM relative to wind speed, indicating improved energy efficiency. The turbine inspired by the cicada, bee, and wasp achieved the highest RPM, followed by the mosquito and fly-inspired design. However, the control design exhibited greater durability, suggesting the need for further refinement to achieve optimal designs. The increase in efficiency, however, was accompanied by a reduction in overall strength, as the insect-inspired designs exhibited breakage under testing conditions. Parameters such as blade shape, vein design, thickness, and curvature can be manipulated to optimize the designs.

Cognet et al. [94]. introduced a wind turbine design, drawing inspiration from biological systems such as insect flight and plant adaptation to wind. The experimental results revealed an increase of approximately 35% in energy yield compared to rigid-bladed counterparts. Based on experiments with the painted lady butterfly (Vanessa cardui), computational models were used to analyze the effects of these wing deformations on aerodynamic performance in a study by Zheng et al. [95]. They investigated the impact of time-varying wing-twist and camber on the aerodynamic efficiency of butterflies during forward flight. Compared to most flying insects, Vanessa cardui is large and has broad wings, with the forewing and hindwing functioning as a single surface (Table 3). The study reveals that the observed butterfly wing outperforms flat-plate wing models (non-deforming) in terms of usable force production and lift-to-power ratio, with at least a 29% and 46% increase, respectively.

Compared to most other insect and bird species, dragonflies have a unique and superior flight performance. As well as gliding for long periods of time, they are also capable of hovering and changing directions quickly. In low wind speeds, dragonflies are distinguished by their agility and aerodynamics [96]. A pair of individually regulated forewings and hindwings gives them their fast-flying ability [97]. Their wings, comprising a complex architecture of membranes and veins, are made of nanocomposite materials, contributing significantly to their functional properties.

Yossri et al. [96] investigated the potential of bio-inspired designs for SWTs using models based on bird and insect wing geometries, specifically the albatross, golden eagle, and dragonfly, with a focus on the performance in low wind speeds (up to 4 m/s) and their ability to handle aerodynamic stresses.

**Figure 12:**
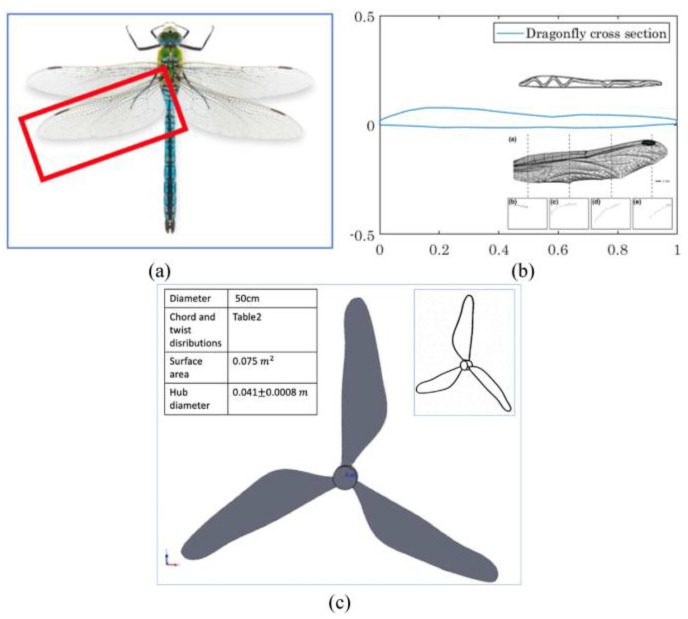
Dragonfly inspired blade design geometric characteristics [96]: (**a**) geometry of wing as found in nature, (**b**) airfoil profile, and (**c**) bio-inspired designed turbine [96]. Reprinted with permission from [96]. Copyright © 2024, Elsevier.

In their exploration of SWT designs, Yossri et al. investigated the unique structure of the dragonfly wing, which distinguishes it from the more common smooth or simply cambered wings of other birds. The dragonfly wing is characterized by a thin, corrugated cross-sectional pattern, a key feature contributing to the wings’ ultra-lightweight and stability. The airfoil profile for the dragonfly model was created based on the method outlined in [98], which involves connecting the maxima and minima along the corrugated pattern of the wing’s cross-section (Figure 12). In the comparative study of bio-inspired models, the dragonfly design, with its lower power output of 2.2 W and torque under 0.11 N m, was noted for its structural efficiency, while the golden eagle-inspired turbine emerged as the most efficient, achieving a 13% C_P_ and generating 4.5 W with 0.21 N m torque. Although the golden eagle design had superior aerodynamics, it faced higher stress levels, unlike the dragonfly design, which effectively reduced stress on the blades.

Prathik et al. [99] investigated the enhancement of VAWT performance through bio-inspired blade designs inspired by natural structures like the maple seed leaf and the eagle wing, but the most significant advancements were seen with the corrugated dragonfly vein FX 63-137 foil.

This dragonfly-inspired design outperformed traditional cambered foils and the standard FX 63-37 model, demonstrating improved efficiency and power output. The maple-wing combined blade structure, incorporating winglet edge tips and the corrugated dragonfly vein foil, showed enhanced lift-drag ratios and higher C_P_.

The significant result of the study was the Corrugated FX foil-embedded maple-wing blade with added back-edge winglets, which showed a substantial improvement over the conventional FX-63137 foil blade. This design achieved a 21% increase in power efficiency and a 39% rise in rotary torque in low-wind-speed conditions (5 m/s and TSR of 4), according to simulations.

In their study, Mulligan [100] investigated the potential of modifying small wind turbine blades by focusing on two key modifications: spanwise corrugations, mimicking a dragonfly’s wing structure, and flexible blades, inspired by the adaptive shape-morphing of bird and insect wings. The research concludes that these innovative turbine designs, especially corrugated airfoils, and bamboo laminates, can enhance performance, particularly in areas with limited facilities. Corrugated airfoils effectively delay stall and maintain lift-to-drag ratios, while bamboo offers a sustainable, accessible alternative to fiberglass, potentially extending blade lifetimes with comparable performance.

Table 4 highlights key features of studies inspired by insects, summarizing research challenges and the advantages of bio-inspired solutions.

## 6. Aquatic Inspirations in Wind Energy

The principles derived from the adaptations and designs of aquatic organisms and creatures have become a compelling source of inspiration for advancing wind energy technologies. The utilization of biomimetic insights from marine life to enhance the performance and sustainability of wind turbines has been investigated repeatedly to shed light on the potential transformative impact of this interdisciplinary approach in renewable energy production.

### 6.1. Fish Schooling

Migratory birds and fish have demonstrated enhanced endurance when traveling in groups, benefiting from their precise positioning to harness vortices generated by their fellow travelers. This natural phenomenon has been explored for its applicability in wind turbines, particularly favoring the vertical axis configuration [101].

In their study, Tescione et al. [102] utilized stereoscopic particle image velocimetry (PIV) to analyze the wake and vortices of a two-bladed H-rotor, notably observing the rapid dissipation of its cycloidal wake just 1.5 rotor diameters downstream. This led to the formation of larger vortical structures, a phenomenon that underpins the VAWT fish-schooling configurations in wind energy.

Whittlesey et al. [103] investigated the design of VAWT farms, drawing inspiration from fish schooling. While HAWTs in wind farms typically suffer reduced power coefficients due to close turbine proximity, VAWTs may experience less decrease or even increases in power coefficients when placed closely, enhancing power output per land area. Using a potential flow model based on fish schooling vortices, their research revealed substantial increases in array performance coefficients, surpassing HAWTs by over an order of magnitude for the same land area.

### 6.2. Humpback Whales

A specific biomimetic innovation, the tubercles found on the leading edge of humpback whale flippers, has garnered significant interest from both industry and the public [104,105,106,107,108,109].

The humpback whale (Megaptera novaeangliae) belongs to the Balaenopteridae family, a species of baleen whale. This remarkable mammal has thrived for approximately 55 million years [110]. The whales typically reach lengths of approximately 15.6 m and weigh around 34 tons [111]. For over a decade, it has been observed that the fins of baleen whales possess remarkable swimming capabilities and maneuverability, enabling them to capture prey effectively. A distinctive feature found on the leading edge of their flippers is the presence of large, rounded tubercles, which are unique structures in the natural world.

In 1979, Jurasz and Jurasz first noticed tubercles on the flippers of humpback whales and the remarkable agility of these large creatures in catching prey [112]. Past practical observations, including those by Hain et al. [113], have shown that the humpback whale’s unique feeding action necessitates a high lift force. According to Weihs [114], the lift force generated is inversely proportional to the whale’s turning radius, indicating that tighter turns are required to produce the necessary lift force.

Experimental studies using models of these structures have demonstrated that they cause a delay in the angle of attack of a blade, resulting in an increased maximum lift and reduced drag. This unique characteristic of tubercles on the leading edges of blades has potential applications in the design of watercraft, aircraft, ventilation fans, and wind turbines [105,115,116,117,118,119,120,121].

Frank Fish, a marine biologist, first initiated the initial research on the presence of bumps on the flippers [110,115,122,123,124,125,126,127,128,129,130,131]. This research led to the development of numerous research articles. Subsequently, Watts and Fish obtained a patent for this technology and established a company called ‘Whale Power’ dedicated to the development of wind turbine blades [132]. The incorporation of tubercle design on the blades has resulted in a 25% increase in airflow compared to conventional wind turbine blades, leading to a 20% boost in energy production [133,134].

Fish et al. reported that tubercles play a role in the humpback whale’s maneuverability [127] and designed wind turbine blades with tubercles based on this inspiration [123]. Wind tunnel tests conducted by Fish’s group showed that blades with tubercles exhibited an increase in the angle of attack, from 11 to 178 degrees, before stalling.

The presence of tubercles on the flipper of the humpback whale also allows for a decrease in drag, resulting in reduced energy consumption during turning maneuvers. This finding suggests that wind turbine blades with tubercles could potentially reduce drag and improve energy efficiency.

According to [115], the addition of leading-edge tubercles on turbine blades has been shown to increase energy generation [105]. Field trials on a 35 kW, variable-pitch wind turbine with tubercle blades by WhalePower Corp. showed improved electrical generation, especially at moderate winds, compared to standard blades. Murray et al. also found tubercle blades effective in marine tidal turbines at low flows [121], indicating their superior performance over smooth-edged blades.

As reported by Zhang et al. [135], the addition of tubercles, despite promoting boundary layer separation under experimental flow conditions, resulted in enhanced power output compared to unmodified blades, especially for stall-regulated turbines operating in wind speeds ranging from 10 to 20 m/s. They also investigated the aerodynamic characteristics of bionic wind turbine blades with sinusoidal leading edge based on a three-dimensional Reynolds-averaged Navier–Stokes simulation [136]. The impact of a single leading-edge protuberance (LEP) on a NACA 634-021 airfoil was explored by Cai et al. [137].

Gopinathan et al. [138] conducted computational comparisons between symmetric NACA 0015 and asymmetric NACA 4415 airfoils, both modified with identical leading-edge tubercles. Their findings revealed that tubercles effectively delay stall occurrence in airfoils.

In a further modification of the tubercled blade design, Ibrahim et al. introduced tubercles on the trailing edge of wind turbine blades [139]. This approach contributed to the stabilization of turbine performance by mitigating turbulence in the wake.

The implementation of the tubercle effect serves multiple purposes, including mitigating stall, minimizing tip vortex, and altering the flow, thereby decreasing flow-induced vibrations and noise generation. This noise, characterized by a high-pitched whistle, has been linked to various applications such as wind turbines, gliders, small aircraft, and fans [140].

Research conducted by Hansen et al. [140,141] demonstrated the potential for suppressing tonal noise by incorporating tubercles into a propeller design. The effective reduction of tonal noise was observed to be more pronounced with tubercles characterized by larger amplitudes and smaller wavelengths. Employing numerical simulations to investigate the interplay between tubercles and airfoil–gust interaction noise, Lau et al. noted an enhancement in noise reduction when tubercles were present [142]. Likewise, several computational studies, including those by Kim et al., Turner et al., and J. Wang et al. [143,144,145], have underscored the role of tubercles in noise reduction by modifying the flow field. J. Wang et al. specifically reported a noise reduction of 13.1–13.9 dB with minimal impact on the drag coefficient in a large eddy simulation involving a NACA 0012 foil equipped with tubercles and trailing-edge serrations [145]. Clair et al. and Polacsek et al. conducted investigations into the tubercle effect on turbofan blades, revealing a 3–4 dB reduction in tonal noise without compromising aerodynamic performance [146,147].

Lv et al. [148] investigated the feasibility of reducing infrasound emissions from existing wind turbine blades using a biomimetic technique. The proposed technique was inspired by the leading-edge tubercles on the fin of humpback whales, the trailing-edge profile of bird wings, and the strips on the body surface of beetles. These biomimetic features are known to reduce vortex shedding and resistance.

The results from both the numerical and experimental studies demonstrated the successful suppression of shedding vortices behind the blade using semi-cylindrical rings. As a result, both infrasound and the overall sound pressure level generated by the blade were significantly reduced.

During an outdoor trial, a wind turbine equipped with nine blades was affixed to a vehicle to manipulate wind velocities ranging from 1 to 7.5 m per second, evaluating the tubercles’ efficiency [149]. It was observed that the tubercled blades exhibited a power increase of 16–30% at wind speeds between 2 and 6.5 m per second. Additionally, Gupta et al. noted that tubercled blades outperformed their straight counterparts by sustaining power generation even during stall conditions [150]. In their study, Abate et al. demonstrated that the strategic placement of tubercles, spanning from 95% of the blade’s span to the tip, resulted in a calculated 10% rise in annual energy production at a wind speed of 10 m/s [151].

Van Nierop et al. [152] investigated the aerodynamic properties of bumps on the leading edge of humpback whale flippers and their effect on stall delay. Through wind tunnel experiments, they observed that these bumps caused a more gradual stall and an increase in the stall angle of attack. The aerodynamic effects of leading-edge modifications inspired by the tubercles on humpback whale pectoral flippers, and their potential to control flow and improve stall characteristics was studied by Rostamzadeh et al. [153]. However, their findings suggest that in turbulent flow regimes, the unmodified foil may offer better lift performance post-stall compared to the tubercled model. In their study, Post et al. [154] explored the hydrodynamic performance enhancements of the tubercles on the leading-edge of humpback whales’ pectoral flippers. They demonstrated that sinusoidal leading-edge wings could mitigate the dramatic lift loss typically caused by stall, instead producing a gradual decrease in lift and achieving up to 25% higher lift in the post-stall regime.

A parametric study to investigate the effect of leading-edge tubercles on the performance of a three-bladed Darrieus VAWT was conducted by Prakash et al. [155] with a focus on reducing wake formation and enhancing the separation length, inspired by the tubercles found on whale fins. In addition, Mishra et al. [156] focused on assessing the impact of leading-edge tubercles on Darrieus-type VAWTs, using computational fluid dynamics and experimental methods to compare them with standard blade turbines. The main objective was to establish an experimental setup to contrast the performances of turbines with and without these tubercles.

Hassan et al. [157] undertook a systematic investigation on the power enhancement of VAWTs through the utilization of leading-edge tubercles inspired by humpback whale flippers. The study explores the relative influence of tubercle design variables and their geometric trends on the power performance of VAWTs. The study provides guidelines for selecting tubercle parameters at different TSRs and emphasizes the importance of considering both performance enhancement and economic aspects in the design process. In their study, Fan et al. [158] conducted a comprehensive hydrodynamic analysis of airfoils with leading-edge tubercles, inspired by humpback whales, at a Reynolds number of Re = 5 × 10^5^. The application of leading-edge protuberances on the humpback whale’s pectoral fin to improve the aerodynamic performance of wind turbines was investigated by Lin et al. [159]. Dimensional analysis revealed that at a 5-degree pitch angle, the C_p_ and the TSR experienced increases of 17.67% and 13.42%, respectively. Additionally, the study found that these modifications stabilized the wind turbine’s output power and increased the average power output. Lobo et al. [160] studied the aerodynamic benefits of incorporating tubercle designs, inspired by humpback whales’ flippers, into wing-like structures such as wind turbines and marine propellers. The study aimed to assess how tubercles affect the performance of a finite wing, using a six-digit NACA 634-421 cambered airfoil, similar to that of a humpback whale’s flipper. Yasuda et al. [161] focused on enhancing the performance of the NACA0012 wing in the low-Re region, specifically within the range of 10,000 ≤ Re ≤ 60,000, using a LEP. The study aimed to understand how varying Re affects the effectiveness of the LEP. The conclusions drawn from this research indicate that airfoils equipped with LEP offer a robust and passive method of separation control in low Re environments, regardless of variations in Re and angles of attack. The results suggest that LEPs could be highly effective as separation control tools in low-speed and small-sized fluid machines, as well as in fluid machines that experience environmental changes during operation.

Mckegney et al. [162] investigated the aerodynamics of a NACA-0021 airfoil with leading-edge tubercles, inspired by biomimetics and humpback whale flippers, to potentially enhance blade design. Tested in low-speed wind tunnel, the study compared this tubercle airfoil’s lift and stall angle with a standard airfoil, revealing a possible 115% increase in post-stall lift and indicating reduced drag.

A summary of studies inspired by marine life with a focus on key features, research challenges, and the benefits of bio-inspired solutions are presented in Table 5.

## 7. Insights and Innovations from Feathered Inspiration

Birds’ wingtip feathers are bent up and separated (like fingers of spreading). This illustrates nature’s solution to reducing drag and enhancing lift [163].

Birds’ wings and wind turbine blades share similar functions [164,165,166], and bionic research has revealed that airfoils inspired by birds, such as the seagull airfoil and those based on the leading edge of long-eared owl wings, exhibit superior aerodynamic performance [167,168]. Birds’ wing leading-edge (LE) profiles have sparked significant interest in aerospace engineering. These wings exhibit flexibility and can adjust their angle of attack during flight maneuvers, but their leading edges typically maintain a consistent spanwise distribution. In cases where bird wings flex during flight, they often feature a swept-back angle, as observed in Videler et al.’s research [169]. This configuration creates a stable LEV, enhancing lift and influencing the bird’s flight dynamics [170]. While engineering adaptations inspired by bird flex wings have been applied to flight wings [171], their integration into wind turbine blade design has been limited.

Following World War I, the French aeronautical engineer G.J.M. Darrieus introduced a VAWT that featured airfoil-shaped blades. His innovative design was granted patents in France in 1925 and in the United States in 1931, drawing inspiration from the aerodynamics of birds’ wings, emphasizing biomimicry in his approach: “It is thus possible to give these blades a streamline section analogous to that of the wings of birds, that is to say, offering the minimum resistance to forward movement and capable of converting into mechanical energy the maximum available amount of energy of the fluid by means of the useful component of the traverse thrust which this section undergoes” [172]. The patent covered two major configurations: curved and straight blades which have evolved into several variations [101].

**Figure 13:**
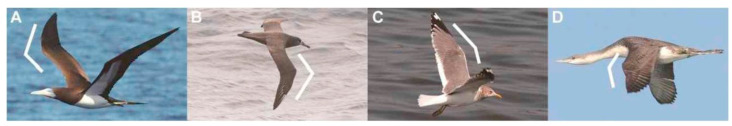
Flexed wings of flying birds in flight. (**A**) Brown booby, Sula leucogaster. (**B**) Black-footed albatross, Diomedea nigripes. (**C**) Common gull, Larus canus. (**D**) Red-throated Diver, Gavia stellate. Photos by courtesy of Yoshiya Odaya (Abiko City Museum of Birds) [173]. Reprinted with permission from [173]. Copyright © 2024, Elsevier.

Ikeda et al. [173] proposed a biomimetic blade design for SWTs, inspired by the robust aerodynamic force production and stable flight of bird wings (Figure 13). The aim was to enhance the robustness of aerodynamic performance by achieving a high integral C_p_ across a wide range of tip-speed ratios.

Bird’s extended feathers, also known as winglets, are another example of bio-inspiration sources [174]. Winglets are recognized for their ability to generate lift and minimize the formation of large vortices [174,175].

Reddy et al. [176] extensively investigated on the design optimization of wind turbine blades with bladelets, aiming to enhance the power output of horizontal-axis wind turbines.

The findings of the study demonstrated that incorporating bladelets on wind turbine blades can lead to an increase in the coefficient of power at off-design conditions while minimizing the penalty on thrust force.

Prathik et al. [99] conducted a design analysis of a VAWT blade using biomimicry techniques. The study aimed to enhance the turbine’s performance and efficiency by incorporating modified turbine blades inspired by biological features such as the eagle wing, maple seed leaf, and corrugated dragonfly vein.

### Owl, Guillemot, Seagull, Albatros, Stork, and Golden Eagle

The aerodynamic prowess exhibited by many owl species during flight and hunting can largely be attributed to their unique airfoil design. Owls require a significant amount of lift to glide silently and evade detection by their prey. As a result, the airfoil structure of owl wings has been extracted and applied in the design of wind turbine blades to enhance their performance [177].

Graham [178] conducted a comprehensive analysis of owl wings, highlighting the distinctive characteristics exhibited by these avian creatures. The study revealed several remarkable features of owl feathers, including a comb-like structure along the leading edge, a fringe-like arrangement along the trailing edge, and soft down feathers on the wings and legs. These unique feather structures enable owls to glide and fly silently, setting them apart from other birds [177]. Ito [179] studied the aerodynamic influence of leading-edge serrations on an airfoil at low Reynolds numbers. The researchers focused on the distinct fine serrations observed on the leading edge of owl wings, which contribute to the reduction of flapping sound. In addition to their sound-damping effect, the authors explored the impact of these serrations on the aerodynamic characteristics of the wing. The authors reported that the presence of serrations enabled the airfoil to maintain lift force at larger angles of attack compared to the prototype wing, particularly at low Reynolds numbers.

Rao et al. investigated the intricate interplay of aerodynamics and acoustics, particularly concerning owl-inspired wing features, prominently leading-edge serrations [180]. Taking inspiration from the natural phenomenon of owls achieving near-silent flight due to their unique wing morphologies, this multidisciplinary study integrated cutting-edge techniques, encompassing Large-Eddy Simulations (LES), Particle-Image Velocimetry (PIV), and controlled wind tunnel experiments. In another study [181], Rao et al. investigated the aerodynamic performance and noise reduction capabilities of owl-inspired leading-edge (LE) serrations in airfoil designs. The research underscores that the effectiveness of LE serrations in flow control and noise reduction depends on how they influence LE vortex breakdown and laminar-turbulent transition, which varies between low and high Res.

A bionic approach was employed in a study by Tian et al. [168] to design wind turbine blade airfoils based on the morphology of the long-eared owl’s wings. The study showed that a bionic airfoil inspired by the long-eared owl’s wing significantly enhanced lift coefficient and stalling performance in wind turbine blades. Tests indicated that this bionic blade achieved an efficiency increase from 12% to 44% over standard blades, due to a larger pressure difference generating stronger lift. Bodling et al. conducted a numerical investigation on the aerodynamic and aeroacoustics performance of airfoil geometries inspired by the down coat of the night owl [182]. The study aimed to understand the mechanisms behind the observed noise reduction at the trailing edge of such bio-inspired designs. The study concluded that the owl-inspired airfoil with inlets showed promising potential for noise reduction. The investigation by Chen et al. [177] focused on the bionic coupling design and aerodynamic analysis of HAWT blades. The study aimed to optimize the design of wind turbine blades by drawing inspiration from the flight characteristics of owls.

The resulting bionic-coupled wind turbine blades exhibited higher lift, torque, and power generation compared to the standard blades, as the authors stated.

In their research, Zhao et al. [183] developed an optimal design method for airfoil geometries, drawing inspiration from the silent flight mechanisms of owl wings, specifically focusing on trailing-edge serrations as a means of controlling aerodynamic noise in wind turbine blades. While previous studies have indicated that adding serrations could negatively impact an airfoil’s overall aerodynamic performance, the study aimed to balance these effects by integrating the fundamental parameters of serrations into the design process. The aerodynamic performance of biomimetic turbine blades inspired by wings of the Common Guillemot species (*Uria aalge*) in the context of ocean current power generation was investigated by Montoya et al. [184].

Hua et al. [167] conducted a comparative analysis of the aerodynamic performance of the seagull and NACA 4412 airfoils for SWTs operating under low-Reynolds conditions. In another study, Hua et al. [185] investigated the design and performance analysis of bionic wind turbine blades. Drawing inspiration from the excellent aerodynamic performance of seagull wings, three types of bionic blades were designed using bionic wing types and configurations, based on the blade element theory of wind turbine blade design.

Experimental wind blow tests corroborated these findings, revealing that the bionic blades achieved lower starting wind speeds and higher rotational speeds compared to the standard blades. Specifically, the bionic blade with a total improved airfoil exhibited an 8% increase in rotational speed, the bionic blade with a partially improved airfoil displayed a 10.2% increase, and the configuration improved blade demonstrated a 6% increase, all relative to the standard blade, at the same wind speed. In their research, Qiao et al. [186] delved into the aerodynamic performance of airfoils based on the seagull’s wing design, particularly focusing on applications for SWTs operating under low-Reynolds conditions where air viscosity significantly impacts blade performance. The research concluded that the seagull airfoil is particularly well suited for the blade tips of SWTs.

Albatross and stork, renowned for their migratory feats and graceful flight over open seas, serve as natural inspirations for the design of HAWTs tailored for low wind velocity applications. Robles et al. [187] investigated the aerodynamic design and performance of HAWTs featuring airfoils inspired by these avian marvels. In the context of wind power as a renewable energy source, capable of reducing carbon emissions and offering cost-effective electricity generation, this the emphasized its potential to attain efficiency levels close to 50%. While the investigation operated on a micro-scale, the authors highlighted the viability of albatross and stork blade designs for modern wind turbines, particularly in achieving C_p_ exceeding 0.4 or 40% efficiency at higher TSRs.

**Figure 14:**
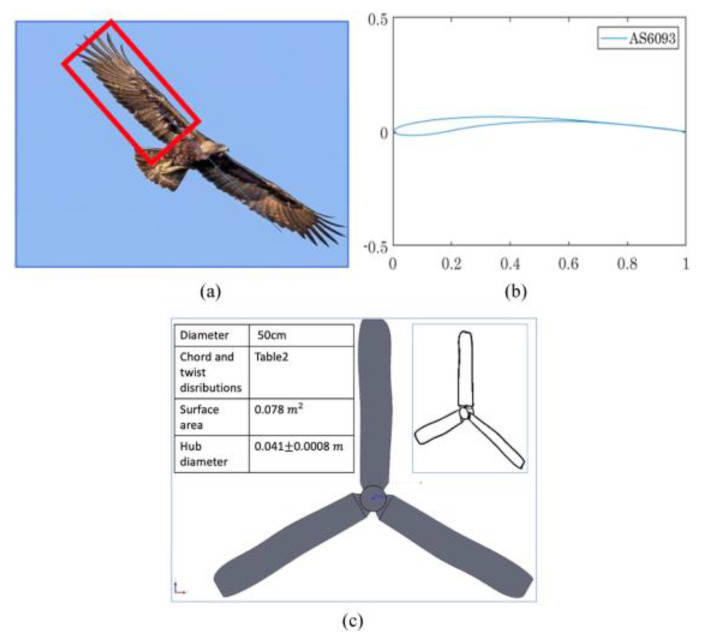
(**a**) Geometry of wing as found in nature, (**b**) airfoil profile, and (**c**) bio-inspired designed turbine [96]. Reprinted with permission from [96]. Copyright © 2024, Elsevier.

Yossri et al.’s research [96] included a focus on the golden eagle’s wing geometry for SWT design. The golden eagle is known for its exceptional flight abilities, including hovering, perching, rapid flight, and high maneuverability during hunting, as described in [188]. These capabilities are attributed to its adaptive airfoil shape, allowing for swift changes and control of aerodynamic loads to suit various flight needs. The golden eagle’s wings, typically forming an obtuse angled “V” in flight with distinctive trailing edges resembling secondary feathers [188], inspired the turbine blade design. The golden eagle-inspired design, as presented in Figure 14, aimed to provide flexibility and adaptability in turbine performance under varying wind conditions. The study [188,189] provided detailed wing geometries and distributions, contributing to a more efficient and adaptable turbine design.

**Figure 15:**
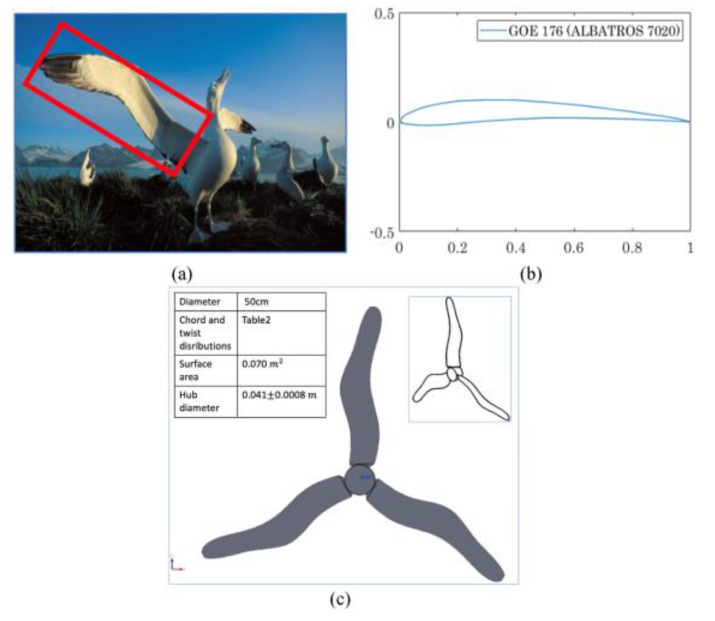
(**a**) Geometry of wing as found in nature, (**b**) airfoil profile, and (**c**) bio-inspired designed turbine [95].

The albatross, another focus of Yossri et al.’s study, intrigued researchers with its unique flight performance, particularly in dynamic soaring. Unlike other birds and engineered flying vehicles, albatrosses gain airspeed and height while gliding into the wind, achieving speeds greater than the wind itself. Studies have shown that albatrosses can reach speeds of 30 km/h in wind speeds not exceeding 13 km/h [96]. This ability to generate high aerodynamic lift made the albatross an attractive model for increasing the lift potential of SWTs. Albatross wings, characterized by a prominent mid-span flexion and a wider trailing edge compared to the golden eagle, inspired the turbine blade design presented in Figure 15. The emphasis on wing flexion has been linked to increased aerodynamic lift in numerous studies [173,190]. The albatross wing geometry, therefore, offered a novel approach to SWT design, focusing on maximizing lift and aerodynamic efficiency. In conclusion, Yossri et al.’s study demonstrated the potential of bioinspired SWT designs, particularly those inspired by the golden eagle and the albatross, for efficient operation at low wind speeds and TSRs.

Table 6 presents the essence of studies inspired by birds, which emphasizes the key features, research challenges, and the advantages of bio-inspired solutions.

## 8. Natural Composite Materials

In the design of wind turbine systems, the selection of materials is a critical factor. The properties of materials used in constructing wind turbines significantly influence their efficiency, durability, and overall performance [191]. These materials must withstand diverse environmental conditions, manage mechanical stresses, and contribute to the aerodynamic efficiency of the turbines. Consequently, advanced composites, metals, and innovative alloys are often chosen for their strength, lightweight nature, and resistance to wear and corrosion. Materials found in nature with unique properties have paved the way for biomimetic materials—synthetic counterparts emulating these characteristics. For instance, spider silk, known for its strength and lightness, has led to the development of synthetic spider silk for textiles and biomedical devices [192]. Nacre, found in mollusk shells, has inspired synthetic nacre for protective coatings and biomedical implants due to its toughness [193]. Additionally, the study of natural fibers such as bamboo, flax, and hemp has influenced the development of sustainable and eco-friendly composites. These materials offer advantages in terms of low weight, high strength, and biodegradability, making them ideal for applications in the construction, automotive, and aerospace industries.

Biomaterials like bone, strong, lightweight, and self-repairing, have inspired synthetic bone grafts and implants [192]. Engineers have developed synthetic composites inspired by the hierarchical structure of bone, incorporating materials like polymers and ceramics to create lightweight, yet strong, materials for various applications, including aerospace and medical implants.

### Insights from Bone Structures and Nacre

Aage et al. [194] presented a computational morphogenesis tool that allows for the design of structures with giga-voxel resolution, enabling unprecedented insights into optimal material distribution. The tool was applied to the design of a full-scale airplane wing, resulting in an optimized wing structure that exhibits remarkable similarity to naturally occurring bone structures found in bird beaks. The results highlighted the potential of computational morphogenesis for achieving optimal designs with enhanced structural performance and reduced environmental impact.

The study presented by Kaminski et al. [195] focused on the development and ground testing of a 1% scaled wind turbine blade.

The researchers used additive manufacturing and a bio-inspired structural design approach to optimize the blade’s mass and stiffness. The design was influenced by the efficient internal structure of bone, which adapts and grows according to applied loads, following Wolff’s law. By incorporating lightweight bio-inspired morphology and carbon fiber reinforcements, the final blade design achieved significant gravo-elastic scaling performance. The use of additive manufacturing, bio-inspired designs, and carbon fiber reinforcements enables the production of lightweight yet structurally efficient blades.

Nacre, also known as mother-of-pearl, is renowned for its remarkable mechanical properties, notably its exceptional toughness. Consequently, it has emerged as a compelling model for the enhancement of tough synthetic ceramics [196,197,198,199]. Notably, abalone nacre exhibits a work of fracture approximately 3000 times greater than that of an individual crystal of the pure mineral [200]. Extensive prior research has elucidated that the distinctive microarchitecture of nacre primarily governs its mechanisms of strengthening and toughening [201,202,203,204].

Mishnaevsky et al. investigated a new concept for developing sustainable wind turbine blades, focusing on bio-inspired design with engineered adhesives. Their study addressed two primary challenges in the wind energy sector: the need to enhance the durability of wind turbine blades, which often degrade over several decades, and the growing demand for recyclability and sustainability in blade design.

The authors highlighted that most common wind turbine blade degradation mechanisms are rooted in interface degradation, including issues like coating detachment, buckling, and adhesive joint degradation. To address these challenges, the proposed concept involves the development of bio-inspired dual-mechanism-based interface adhesives, combining the mechanical interlocking of fibers and chemical adhesion. The study concluded that such adhesives could lead to wind turbine blades with longer lifetimes, reduced maintenance requirements, and improved recyclability, addressing key challenges in the field of wind energy.

Table 7 underlines key features of studies inspired by natural composites, summarizing research challenges and the advantages of bio-inspired solutions.

## 9. Fibonacci Sequence: Implications for Wind Energy Innovation

The Fibonacci sequence and the golden ratio are intricately linked in the construction of geometric patterns, particularly the golden rectangle and spiral. In essence, the behavior of the Fibonacci sequence is often described as quadratic recursion [206]. This sequence can be generated for any integer *n* ≥ 2, where each number formed is the sum of the preceding two numbers (Fn=Fn+1+Fn−1). The Fibonacci sequence frequently manifests in geometry and patterns observed in nature, such as the arrangement of sunflower florets, the spiral of a pinecone, the structure of a nautilus shell, and more. The Fibonacci spiral, also known as the logarithmic spiral, emerges through the growth factor related to the golden ratio. The Fibonacci sequence finds extensive applications across various fields of study, showcasing asymptotic behavior that can be described through linear recurrence [207].

El-Sheikh [208] explored a novel wind turbine blade design aimed at addressing the challenges associated with transporting large-scale wind turbines. These challenges primarily stem from road design, terrain conditions, and logistical constraints when moving blades to windy sites. Drawing inspiration from nature’s finger and the Fibonacci sequence, the study introduced a blade with the capability to fold, enhancing maneuverability and simplifying transportation routes while reducing costs.

The mathematical interpretation of Savonius wind turbine blade geometry, specifically focusing on its semicircle shape, was investigated by Ashwindran et al. [209]. The study delved into the √2 conjecture as it relates to circle construction, along with its connections to the Fibonacci and Pythagoras spirals concerning √2, √2 + 1, and √2 + 2. The research demonstrated that the √2 conjecture can effectively determine the geometric properties of circles and spirals. The results highlighted an enhancement in Cm for the proposed blade shape, showcasing a 7.2% improvement at λ = 0.59 and a 4% increase at λ = 0.94 when compared to conventional Savonius wind turbines (SWT). Damota et al. researched the performance of a nature-inspired shape for a VAWT [38,210]. The study focused on improving the efficiency of a Savonius-type turbine by utilizing a blade profile based on the Fibonacci spiral. They introduced the Fibonacci shape to explore the impact of various parameters such as the number of blades, aspect ratio, overlap, separation gap, and twist angle. In another study, the authors aimed to investigate the influence of these parameters on the performance of the blade profile. Remarkable improvements exceeding 30% in average power were achieved by optimizing these parameters. The average C_P_ and C_T_ were improved by 14.1% and 13.5%, respectively, with the Fibonacci blade profile. The findings highlight the potential of the Fibonacci-inspired design for enhancing the performance of VAWTs.

Some authors of this study have suggested a bio-inspired shape for marine propulsors in their previous works, showing improvements in the performance of conventional propellers in specific applications [211,212,213,214].

The key features of studies inspired by the Fibonacci sequence, the research challenges, and the advantages of bio-inspired solutions are summarized in Table 8.

## 10. Discussion

It is evident from the reviewed research that biomimetics has played a significant role in addressing various challenges and optimizing the performance of wind turbines. The sources of inspiration can be categorized into different biological realms, each offering unique insights and solutions (Table 9).

One of the prominent sources of inspiration is the humpback whale tubercles, which have been extensively studied. The tubercles on humpback whale flippers have been a noteworthy source of inspiration, extensively influencing engineers to enhance wind turbine blade efficiency, performance, and noise reduction.

Birds, specifically the wings of owls, have inspired research in aerodynamics and silent flight. These studies have implications particularly in the context of noise reduction in wind turbines. These studies have shown the potential of biomimetic design to mitigate the noise generated by wind turbines, contributing to reduced environmental impact and enhanced acceptability.

Inspiration from the plant kingdom, particularly seeds like maple and samara seeds, has also gained attention in wind turbine design. This approach draws from nature’s efficiency in seed dispersal mechanisms, translating into innovative blade designs for improved aerodynamics and energy capture.

Insects’ wings, known for their intricate structures and adaptability, have been a source of inspiration for wind turbine design. Researchers have explored how the microstructures and aerodynamics of insect wings can be applied to improve the performance of turbine blades.

Furthermore, the application of biomimetics extends to natural composites like nacre, which has been used to develop adhesives for wind turbine structures. This approach leverages the remarkable mechanical properties of natural composites to enhance the durability and longevity of wind turbines.

The study also discusses research inspired by bone structure, emphasizing the potential of bio-inspired materials and structural designs to address the challenges of wind turbine construction and maintenance.

The Fibonacci sequence, a mathematical concept inspired by natural patterns, has also found its way into wind energy research. This sequence has been utilized to optimize blade shapes and configurations, showcasing the interdisciplinary nature of biomimetics.

Despite the broad range of applicability of biomimetics in various fields of engineering and science, the application of biomimetics in wind energy system design has remained limited in both scope and quantity. These limitations manifest in two key aspects: the restricted variety of biological sources explored for inspiration and the relatively low number of research endeavors dedicated to the application of biomimetics in wind energy.

Firstly, when examining the range of biological sources that have informed biomimetic designs for wind energy systems, it becomes apparent that this diversity is somewhat lacking. While some promising inspirations have been drawn from sources such as the humpback whale tubercles, seeds, birds’ and insects’ wings, and mathematical concepts like the Fibonacci sequence, the overall pool of biological sources remains relatively constrained. Factors contributing to this limitation could include the intricacies of translating biological principles into practical engineering solutions, the availability of experts versed in both biology and wind energy engineering, and the unique challenges posed by the wind energy industry.

Secondly, despite the potential benefits and innovative solutions that biomimetics can offer to enhance wind energy systems, the number of research projects dedicated to this specific domain is noticeably low. This scarcity of research efforts is somewhat surprising given the increasing demand for sustainable and efficient energy solutions and the numerous challenges faced by the wind energy sector. The limited attention afforded to biomimetic approaches in this context may be attributed to factors such as the complexity of interdisciplinary collaboration between biologists and wind energy engineers, the costs and resources associated with conducting experimental investigations, and the overall awareness and prioritization of biomimetics within the wind energy research community.

In conclusion, while the application of biomimetics holds significant promise for advancing wind energy system design, the current landscape reveals both a restricted variety of biological inspirations and a lack of research initiatives in this field. Addressing these issues may unlock untapped potential for harnessing the remarkable efficiencies and innovations found in nature to bolster the performance and sustainability of wind energy systems.

## 11. Conclusions

The synthesis of biomimetics and wind energy represents a promising frontier in the pursuit of sustainable and efficient renewable energy solutions. This review has provided an examination of the diverse research endeavors that have harnessed biomimetic principles to enhance the design, performance, and ecological impact of wind energy systems.

Through a systematic survey of the existing literature, it has become evident that biomimetics offers multifaceted advantages in the wind energy domain. Humpback whale tubercles, subject to extensive study, are utilized to enhance wind turbine blade efficiency and mitigate noise.

Owl wing aerodynamics are drawn upon for the development of silent flight technology in wind turbines, resulting in the reduction of noise pollution and environmental impact. Botanical inspiration from seeds informs the design of advanced blades, contributing to improved aerodynamics and enhanced energy capture. Several researches have been conducted to enhance blade performance, drawing inspiration from the intricate microstructures found in insect wings. Natural composites, including nacre, are employed in the development of robust adhesives, thereby extending the longevity of wind turbines. Bio-inspired structural designs have been explored to address construction and maintenance challenges in wind turbine systems. Furthermore, mathematical concepts such as the Fibonacci sequence are employed to optimize blade shapes, highlighting the multidisciplinary approach of biomimetics in wind energy research.

In conclusion, as we navigate the promising frontier of biomimetics and its integration with wind energy, a future direction that emphasizes the role of biomimetics in sustainable designs is foreseen. Biomimetics has already demonstrated its potential to enhance designs for greater sustainability and environmentally friendly outcomes across various domains. Looking ahead, it becomes increasingly vital to explore the applicability of biomimetic principles in finding innovative solutions for managing decommissioned wind turbines. Exploring what nature offers for repurposing, recycling, or reusing these turbines aligns with the principles of ecological responsibility and sustainability at the heart of green energy initiatives.

With biomimetics illuminating the path forward, we find ourselves at the threshold of a fresh beginning in wind energy, where innovation converges with environmental responsibility to propel us toward a cleaner and more harmonious future. In this collaborative endeavor, the echoes of nature’s wisdom resonate through the turbines, whispering the promise of a greener tomorrow.

## Figures and Tables

**Table 1:** Symbolic representation of inspirational sources.

Plants	Insects	Aquatic Inspirations	Birds	Natural Composite Materials	Fibonacci Sequence
** 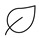 **	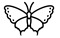	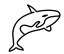	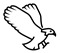	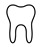	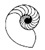
Movement of tree branches and leaves Lotus flower (Nelumbo nucifera) Maple seed Triplaris samara seed Dryobalanops aromatica seed Borneo camphor seed Petrea Volubilis seed Mimosa seed Bauhinia Variegata seed Epilobium hirsutum seed	Cicada Bee Wasp Mosquito Dragonfly	Fish schooling Humpback whales	Owl Guillemot Seagull Albatros Stork Golden eagle	Nacre Bone Structures	Natural finger’s ability to fold Natural geometrical patterns

**Table 2:** The summary of the research findings inspired by the plant kingdom.

Study (Author)	Focus	Key Features	Advantages	Challenges	Source of inspiration
McGarry et al. [58].	Wind energy harvesting	Investigation of energy harvesting from tree movement for powering wireless sensor nodes	A sustainable energy source for sensor nodes in forests; utilizing tree movements which are abundant and constant in forests; Offering an alternative to solar and wind energy in densely wooded areas	Accurate measurement of energy from tree movements is challenging; efficiency and practicality of energy harvesting devices need to be optimized; dependence on variable factors like wind speed and tree properties	The natural and consistent movement of trees in forests
McCloskey et al. [58,59].	Wind energy harvesting	Exploration of piezoelectric elements in artificial plants for wind energy harvesting	Aesthetic and ecological advantages compared to traditional turbines; potential for deployment in residential settings due to less noise and height restrictions	Low power output relative to size and number of synthetic leaves required; challenges in impedance matching and charge production; material limitations and inefficiencies in piezoelectric transduction	The natural motion of plants like cottonwood and cattails
Abdelrahman et al. [63].	Wind turbine blades	Analysis of Nelumbo Nucifera petals’ structure in relation to Betz’s law; comparing standard NACA 2412 and Lotus-inspired blades	Higher efficiency in real-life conditions for Lotus-inspired blades; applicability in small and medium-scale wind energy projects	Manufacturing challenges for larger turbines; optimization of turbine blades at different angles of attack	Nelumbo Nucifera (Lotus)
Lentink et al. [76].	Aerodynamics of Autorotating Plant Seeds	Study of lift generation in autorotating seeds of maples and hornbeam; analysis of LEV in autorotating seeds; comparison of seed aerodynamics with those of hovering insects, bats, and possibly birds	High lift generation during descent; prolonged airtime compared to nonautorotating seeds; convergent aerodynamic solution with certain animals	Understanding the complex interplay of inertial and aerodynamic properties in autorotation; application of findings to practical designs or technologies	Maple and hornbeam seeds
Holden et al. [77].	Maple Seed Aerodynamics and Wind Turbine Design	Flow field analysis around a maple seed; comparison of maple seed dynamics to wind turbine blades; physical and geometric properties measured from real maple seed samples	High efficiency in energy conversion	Scaling from small seed size to practical wind turbine dimensions; structural limitations at larger scales	Maple seed (Acer Negundo)
Seidel et al. [74].	Small Wind Turbine Blades	Structural analysis of vertical axis wind turbine blades inspired by maple seeds and triplaris samara seeds; exploration of whale tubercles for design improvement	Potential for improved efficiency in urban settings; increased lift and decreased drag; suitability for small-scale applications	Ensuring structural integrity at higher wind speeds; balancing increased lift with manageable drag; adapting complex biological shapes for practical engineering applications	Maple seeds and triplaris samara seeds
Herrera et al. [78].	Wind turbine blades	Design of a low scale bio-inspired wind turbine blade; Structural and aerodynamic analysis	Improved aerodynamic characteristics; potential for higher energy efficiency at low wind speeds; utilization of composite materials for enhanced strength	Complex manufacturing process; balancing aerodynamic and structural requirements; ensuring consistent quality in composite material production	Triplaris Americana seed
Carré et al. [75].	Wind energy harvesting	Bioinspired design of harvesters that operate at wind speeds from 1.2 to 8 m/s; output power from 41 µW to 81.7 mW; maximum efficiency of 17.8%; C_P_ up to 28.4%	Expanded range of operable wind speeds; high efficiency and C_P_ for its size; potential application in powering Wireless Sensor Nodes (WSNs); small and practical for various environments	Fabrication precision limitations; efficiency improvement at low wind speeds; technological advancements needed for changing blade angles dynamically; improvements in bearing to reduce losses	Maple samaras
Chu et al. [80].	HAWT rotor blade	Comparison with a conventional tapered and twisted blade turbine; analysis includes C_P_, C_T_, torque, and blade root bending stresses	Higher torque and better self-starting ability at low wind speeds; reduction in blade root bending stress; potential for cost-effective manufacturing due to simpler blade geometry	Lower maximum C_P_ compared to a conventional turbine; need for additional research on flexible blades; managing higher C_T_ and bending moments in structural design	Dryobalanops aromatica seed
Chu et al. [82].	Wind turbine blade	FBWT inspired by biomimicry; centimeter-scale design suitable for powering small devices; Comparing the performance of a FBWT with a RBWT at a centimeter scale	Higher power output in FBWT compared to RBWT; improved self-starting capability; flexible blades leading to faster rotation and yawing; effective in low-Reynolds-number regimes	Adapting large-scale wind turbine designs to centimeter-scale turbines; managing aerodynamic behavior at low Reynolds numbers; designing effective airfoils for small-scale turbines	Borneo camphor seed (Dryobalanops aromatica)
Chu et al. [83].	Wind turbine blade	Thin, cambered, bent blade design mimicking nature	Higher peak C_P_ indicating better power efficiency; better self-start capability due to higher starting torques; lower C_T_ at peak power, implying cheaper supporting structures; suitable for low-speed operation, resulting in less noise and environmental impact; potential cost-effective manufacturing	Optimization of geometry for maximum power output; simplification of design for cost-effective manufacturing; validation of CFD results with physical wind tunnel tests	Borneo camphor seed (Dryobalanops aromatica)
Gaitan-Aroca et al. [84].	Wind rotor	Five-bladed rotor with variable pitch angles based on Petrea Volubilis seed	Potential for efficient kinetic energy transformation in wind power generation; biomimicry approach may offer unique aerodynamic benefits; capability to produce power at lower upstream velocities	Need for optimization of blade cross-section and aerodynamic profiles; complexity in computing aerodynamic coefficients due to unique rotor design; large computational resources required for accurate CFD simulation	Petrea Volubilis seed (also known as Queen’s Wreath or Machiguá flower)
Venkataraman et al. [85].	VAWT rotor	Investigation of the stand-still characteristics of a 500 W bio-inspired VAWT rotor, suitable for urban environments, inspired by nature	Potential for early start-up at low wind speeds (as low as 2 m/s); suitability for urban environments with turbulent wind conditions; potential improvement of aerodynamics and efficiency; helical blade structure may result in smoother and quieter operation	Determining the optimal geometric parameters; ensuring that the rotor’s aerodynamic torque exceeds the cogging torque of a typical generator for self-starting capability; adapting bio-inspired designs to efficiently convert wind energy in an urban setting	Mimosa and Bauhinia Variegata seed
Ashwindran et al. [86].	Wind Turbine Blade	Unsteady numerical investigation of a bi-inspired VAWT for offshore regions in Malaysia, with a hybrid blade design inspired by maple seed and epilobium hirsutum	Optimal performance at certain TSRs; high C_P_ results; potential for efficient offshore wind energy harvesting due to minimal environmental impact and low GHG emissions	Managing the wake and vorticity effects on turbine performance at different TSRs; ensuring stability and high moment coefficients in varying wind conditions; adapting the hybrid bio-inspired design for practical offshore wind energy harvesting applications	maple seed and epilobium hirsutum

**Table 3:** Different insects’ mass (g), wing length (cm), wing area (cm^2^), aspect ratio (4R2/S), and wing loading (g/cm^2^) [95].

Insect	Mass (g)	Wing Length (R) (cm)	Wing Area (S) (cm^2^)	Aspect Ratio (4R^2^/S)	Wing Loading (g/cm^2^)
Vanessa cardui	0.29	3.01	11.4	3.18	0.0254
Schistocerca gregaria	2.08	5.33	29.9	3.80	0.0696
Manduca sexta	1.41	4.96	17.4	5.66	0.0812
Drosophila melanogaster	0.001	0.239	0.0382	5.98	0.0262

**Table 4:** The summary of the research findings inspired by insects.

Study (Author)	Focus	Key Features	Advantages	Challenges	Source of inspiration
Cognet et al. [94].	Developing wind turbine blades	Elastic blades that adapt to wind conditions	35% increase in energy yield; Passive adaptation to wind conditions without extra energy input; Enhanced performance range beyond specific working regimes.	Optimizing the balance between blade flexibility and structural stability; Addressing variability in wind conditions and turbine operational regimes; Implementing the flexible blade design in practical wind turbine applications.	Flapping flight of insects and reconfiguration of plants in response to wind
Segev et al. [93].	wind turbine blade designs	Biomaterial, wing-inspired designs for increased RPM and efficiency	Improved RPM and energy efficiency	Reduced overall strength and durability compared to traditional designs	cicada, bee, wasp, mosquito, and dragonfly wings
Zheng et al. [95,96].	Butterfly wing aerodynamics	Analysis of wing-twist and camber effects	Increased force production and lift-to-power ratio	Focused on a single species.	Butterfly wings
Yossri et al. [96].	designs for small-scale wind turbines	Designs based on bird and insect wing geometries	Golden eagle design most efficient; dragonfly design reduces stress	Low power output in some designs	Dragonflywings, and albatross and golden eagle wings
Prathik et al. [99].	VAWT blade	Corrugated Dragonfly vein FX 63-137 foil	Improved efficiency and power output, especially at low wind speeds	Inefficient self-starting, mechanical losses, turbulent wake issues	Dragonfly wings
Mulligan [100].	Modifying wind turbine blades	Spanwise corrugations and flexible blades	Delayed stall, reduced peak stresses	Slight reduction in peak lift-to-drag ratio	Dragonfly wings

**Table 5:** The summary of the research findings inspired by the aquatic life.

Study (Author)	Focus	Key Features	Advantages	Challenges	Source of Inspiration
Tescione et al. [102].	wake and vortices in wind turbines	Stereoscopic PIV to examine wake and vortices of a two-bladed H-rotor.	Swift recovery of the rotor’s wake, substantial vortical structures downstream.	Complexity in capturing and analyzing the unsteady, three-dimensional flow field; Technical challenges in setting up and conducting stereoscopic PIV measurements	Fish schooling
Whittlesey et al. [103].	VAWT farm design.	Potential flow model to assess VAWT spatial arrangement impacts on performance.	Suggests potential increases in power output for VAWTs compared to HAWTs in a given area; demonstrates that VAWT arrays may have smaller spacing without substantial performance decrease.	Accurately capturing complex aerodynamics and vortex interactions in VAWT arrays; ensuring model accuracy with limited field data; addressing three-dimensional effects, turbulence, and vortex shedding	Fish schooling
Fish et al. [127].	wind turbine blades	Tubercles on blade leading edges to increase attack angle and reduce drag	Enhanced performance at low wind speeds, delay in stall angle.	Complexity in Morphological Analysis; Hydrodynamic Modeling and fluid dynamics complexities; Applying biological insights to engineering has its challenges	humpback whale flippers
Zhang et al. [135].	wind-turbine blades	Utilization of small flat delta wings as control units on wind turbine blades, replacing uncontrollable leading-edge tubercles; active control to adjust to various inflow conditions	Enhanced power output at high-speed inflows; reduction of shaft-torque fluctuation from 27.8% to 8.9%; maintenance of power output under design conditions	Managing the complexity of blade design with active control elements; ensuring stable aerodynamic performance under varying conditions; addressing early boundary-layer separation at blade’s suction side	humpback whale flippers
Zhang et al. [136].	wind-turbine blades	Investigation of the aerodynamic characteristics of bionic wind turbine blades with sinusoidal leading edge	Improved shaft torque at high wind speeds; enhanced aerodynamic performance as the blade enters stall; better power output in the outboard segment	Early boundary-layer separation under design conditions; reduction in shaft torque for wavy-blade cases at design wind speeds	humpback whale flippers
Cai et al. [137].	wind-turbine blades	Exploring the impact of a single LEP on a NACA 634-021 airfoil	Provides insights into the complex effects of leading-edge modifications on airfoil performance; high consistency between theoretical models and experimental results	Understanding and accurately predicting the nuanced flow mechanisms induced by protuberances; complexity in integrating experimental observations with theoretical models	humpback whale flippers
Ibrahim et al. [139].	wind turbine blades	Stabilization of turbine performance by mitigating turbulence in the wake.	Enhanced power at lower wind speeds; better performance in severe wind conditions; more stability under unsteady and higher wind velocities	Achieving optimal design configurations that balance improved performance against manufacturing complexities and cost; understanding the precise aerodynamic mechanisms behind the advantages of these designs	humpback whale flippers
Hansen et al. [140,141].	Study of the effect of leading-edge tubercles on airfoil flow and noise	Introduction of sinusoidal modifications (tubercles) to the airfoil’s leading edge; focus on the reduction of tonal noise and overall broadband noise; exploration of the effects of varying the amplitude and spacing of tubercles on noise reduction	Elimination of tonal noise at certain angles of attack; reduction in overall broadband noise surrounding peak tonal noise frequencies; possible enhanced aerodynamic performance	Determining optimal configurations of tubercle amplitude and spacing for effective noise reduction; balancing noise reduction with maintaining or improving aerodynamic efficiency	humpback whale flippers
Lv et al. [148].	Reducing infrasound emissions from wind turbine blades.	Use of semi-cylindrical rings wrapped on the blade; targeted suppression of shedding vortices behind the blade; focus on reducing both infrasound and overall sound pressure level; improvement in the C_P_ of wind turbines	Non-invasive modification to existing blades; cost-effective and easy to implement; reduction of infrasound emissions; potential improvement in wind turbine efficiency	Optimal configuration of semi-cylindrical rings; avoiding negative impacts on blade performance and turbine efficiency	humpback whale flippers
Gupta et al. [150].	aerodynamic performance of innovative wind turbine blade designs	Study different blade designs using NACA 4412 airfoil; comparative analysis of straight swept blade, winglet, tubercle, and slotted blades; emphasis on enhancing power generation efficiency	Increased electrical generation, particularly at moderate wind speeds.	Balancing efficiency and design complexity; ensuring reliability and durability of innovative blade designs	humpback whale flippers
Van Nierop et al. [152].	Wind turbine blades	Analysis of the effects of leading-edge bumps on stall delay; development of an aerodynamic model to explain the observed increase in stall angle; study of lift curve behavior with varying bump amplitude	Increase in stall angle by up to 40% without compromising lift or drag; gradual onset of stall, enhancing control properties	Translating complex biological adaptations into practical engineering designs; addressing potential discrepancies in experimental results and theoretical models	humpback whale flippers
Prakash et al. [157].	Wind turbine blades	NACA0018 blade modification with leading-edge tubercles for a Darrieus VAWT; aerodynamic analysis to assess tubercle impact on separation length and wake reduction; examination of various tubercle design configurations for enhanced blade efficiency	Tubercle profile increases separation length and decreases wake region, potentially reducing drag; improvement in flow reattachment on the blade	Managing the complexity of modeling and computational analysis for tubercle-enhanced blades.; need to evaluate additional parameters like torque for more decisive conclusions	humpback whale flippers
Hassan et al. [143].	Enhancing power performance of VAWTs with leading-edge tubercles.	DoE approach and RSM to assess power performance.	Enhanced power performance under off-design conditions.	Conflicting findings in the literature.	humpback whale flippers
Lin et al. [159].	Improving wind turbine performance using a biomimetic approach	Investigations of modified airfoils and turbine blades	C*p* increased by 17.67%; TSR increased by 13.42%; reduced power output variation; enhanced stability in power generation	Potential challenges in adapting biological structures to engineering designs; ensuring consistent performance under varying wind conditions	humpback whale flippers
Fan et al. [158].	Examining the impact of leading-edge tubercles on airfoil performance	Examining a reference airfoil and two modified airfoils with leading-edge tubercles; utilizing nonlinear shear transformation for tubercle design; analyzing lift and drag coefficients, lift-to-drag ratios; investigating CRVPs for momentum exchange	Enhanced lift coefficient after stall in modified airfoils; smoother and more stable stall process; improved momentum exchange in trailing edge boundary layer	Complexity in understanding the flow control mechanism of leading-edge tubercles; challenges in optimal design and application of tubercles	humpback whale flippers
Mckegney et al. [162].	wind turbine blades	Investigating the effect of tubercles on wind turbine blade aerodynamics with a focus on post-stall lift enhancement and induced drag reduction	Enhanced post-stall lift by 115% indicating reduced induced drag in post-stall; potential for increased efficiency in wind turbine applications	Balancing pre-stall and post-stall performance improvements; optimizing amplitude and wavelength ratios for tubercles	humpback whale flippers

**Table 6:** The summary of the research findings inspired by birds.

Study (Author)	Focus	Key Features	Advantages	Challenges	Source of inspiration
Liu et al. [154].	wind turbine blades for small scale wind turbines	Seagull inspired airfoil design	Higher lift coefficient; Greater scope of working angle of incidence; Higher lift-drag ratio	Adapting bird-inspired airfoil designs to effectively function in wind turbine applications; Ensuring efficient performance across a range of operational conditions.	Seagull wings
Tian et al. [168].	Airfoils for wind turbine blade	Airfoil design based on Long-eared Owl’s wing	Superior lift coefficient and stalling performance; Better pressure difference between upper and lower surfaces.	Effectively replication of the aerodynamic advantages seen in nature.	Long-eared Owl Wings
Ikeda et al. [173].	Blade design for SWTs	Introduction of bird-inspired flexed wing morphology; Robustness Index (Ri) proposal	High integral C_P_ across tip-speed ratios	Adapting blade design to broad tip-speed ratios, Ensuring robust aerodynamic performance under variable conditions	Bird Wings
Reddy et al. [176].	Wind turbine blades with bladelets	3D flow-field analysis, Multiobjective constrained shape design optimization	Increase in coefficient of power at off-design conditions, Minimized penalty on thrust force	Complexity in optimizing bladelet geometry, Balancing multiple objectives in design	Bird’s winglets
Prathik et al. [99].	VAWT Blade Design	Analysis of biostructure blades, Simulation of low wind speeds, Comparison with traditional foils	Enhanced lift-drag ratio, higher coefficient of power	Design complexities in mimicking biological structures	Maple Seed Leaf, Eagle Wing
Chen et al. [177,179].	HAWT Blades Design	Bionic coupling design, non-smooth LE	Increased lift, torque, power generation	Refinement of design parameters	Owl Wings
Ito [179].	Aerodynamics of Serrations	Leading-edge serrations impact	Maintenance of lift force at larger AoAs	Focused on low Reynolds numbers	Owl Wings
Rao et al. [180].	Aeroacoustic Control and Noise Reduction	LEserrations for noise reduction over a broad Re range	Passive control mechanism, reduced noise; improved lift-to-drag ratio, noise reduction	Trade-off between noise reduction and aerodynamics, effectiveness varies with Re	Owl Wings
Bodling et al. [182].	Aeroacoustic Performance	Noise reduction at trailing edge	Reduction in high-frequency noise	Understanding noise reduction mechanisms	Owl’s Down Coat
Zhao et al. [183,184].	Airfoil Design	Optimal design of serrated airfoils	Enhanced aerodynamics and noise reduction	Balancing noise control with performance	Owl Wings (Trailing-Edge Serrations)
Montoya et al. [184].	Biomimetic Turbine Blades	Inspired by bird wings	Higher lift-to-drag coefficients	Sub-optimal blade settings, limited velocity range	Common Guillemot Species
Hua et al. [167]	Bionic Wind Turbine Blades	Design of three types of bionic blades inspired by seagull wings, Blade element theory	Increased blade torque; Favorable aerodynamic performance; Lower starting wind speeds; Higher rotational speeds	Balancing the biomimetic design with aerodynamic efficiency	Seagull Wings
Qiao et al. [186]	small wind turbine blades	Bionic design of wind turbine blades, Numerical simulation of aerodynamic performance, Comparison with traditional airfoil (NACA 4412)	Higher lift coefficient and better stalling performance; Suitability for small-power wind generators; Potential increase in efficiency	Adapting bionic designs to practical engineering applications, Ensuring structural integrity while optimizing aerodynamic performance	Seagull’s Wing
Robles et al. [187].	HAWT Design with bio-inspired airfoils	Airfoils inspired by Albatross and Stork, Low wind velocity optimization	Potential for improved aerodynamic efficiency in low wind conditions; Increased power output at lower wind speeds	Balancing bio-inspired design with engineering constraints; Ensuring structural integrity and long-term durability; Optimizing performance across varied wind conditions	Albatross and Stork
Yossri et al. [96].	Blade designs for low-speed small-scale wind turbines	Golden eagle and albatross-inspired blade designs	High power output and torque, effective in low-wind conditions	Structural integrity and manufacturing complexity	Albatross, golden eagle, dragonfly wing geometries

**Table 7:** The summary of the research findings inspired by natural composites.

Study (Author)	Focus	Key Features	Advantages	Challenges	Source of Inspiration
Mishnaevsky, Jr., et al. [205].	Sustainable Wind Turbine Blades	Bio-inspired design, engineered adhesives; interface control for durability and recyclability	Extended blade lifetime, reliability, and sustainability; strong and detachable adhesives	Preventing blade degradation and failure; development of sustainable, recyclable blades	Biological composites like nacre, shells, skulls/teeth/bones, timber/bamboo
Kaminski et al. [195].	Wind turbine blade	Bio-Inspired Structural Desig; Additive Manufacturing; down scaling the blade while maintaining key dynamic and structural properties	Cost-Effectiveness; Innovative Design Capability; High Fidelity Modeling	Scaling Complexity, Material and Manufacturing Constraints	The internal structure of bones

**Table 8:** The summary of the research findings inspired by Fibonacci sequence.

Study (Author)	Focus	Key Features	Advantages	Challenges	Source of Inspiration
El-Sheikh [208].	Wind turbine blade	Design Inspiration; Blade foldability for easier transport; Modifications to blade Skin and Spar; Flexure hinge design for folding ability; Utilizes corrugated shape in flexure zones.	Enhanced Maneuverability; Reduced Simplifies route scenario and reduces transportation cost; Applicability to Large Blades	Maintaining structural integrity and performance while incorporating folding joints; Manufacturing Complexity; Managing stress distribution, especially at the flexure hinges during folding and operation	Fibonacci sequence and the natural finger’s ability to fold
Ashwindran et al. [209].	Wind turbine blade	Exploration of √2 conjecture in circle and Fibonacci spiral construction; Adapting the conjecture for blade design in DIWT.	Improved moment coefficient; the utility of irrational numbers in the design of complex geometries; Potential for multiple blade curvature combinations.	Ensuring the accuracy and reliability of mathematical conjectures in practical applications; Managing the complexity of CFD simulations for blade design.	Fibonacci sequence and nature’s geometrical patterns
Damota et al. [38,210].	Wind turbine blade; improvement of efficiency in VAWT	Fibonacci spiral-based blade profile; comparison with traditional semi-circular Savonius blade profile; analysis of various parameters like number of blades, aspect ratio, overlap, separation gap, and twist angle for optimization.	Improvement in average C_P_; improvement in average C_T_ compared to Savonius profile	Determining the optimal combination of turbine design parameters; Aligning the turbine design with practical and economic feasibility in urban environments.	Fibonacci sequence

**Table 9:** The investigated natural sources of inspiration and their corresponding applications in wind turbine system design.

Application	Source of Inspiration
Wind turbine design and farm	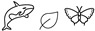
Rotor design	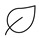
Airfoil design	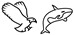
Wind turbine blades	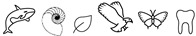
Aerodynamics study	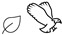
Noise reduction	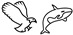
Efficiency & performance	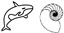
Energy harvesting	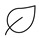
